# Effects of Multisession Anodal Electrical Stimulation of the Auditory Cortex on Temporary Noise-Induced Hearing Loss in the Rat

**DOI:** 10.3389/fnins.2021.642047

**Published:** 2021-07-29

**Authors:** Iván Díaz, Ana Cecilia Colmenárez-Raga, David Pérez-González, Venezia G. Carmona, Ignacio Plaza Lopez, Miguel A. Merchán

**Affiliations:** Instituto de Neurociencias de Castilla y León (INCYL), Universidad de Salamanca, Salamanca, Spain

**Keywords:** corti organ, auditory brainstem responses, quantitative immunocytochemistry, choline acetyl transferase, epidural anodal direct current stimulation, cochlear inflammatory response, cochleotopy

## Abstract

The protective effect of the efferent system against acoustic trauma (AT) has been shown by several experimental approaches, including damage to one ear, sectioning of the olivocochlear bundle (OCB) in the floor of the IV ventricle, and knock-in mice overexpressing outer hair cell (OHC) cholinergic receptors, among others. Such effects have been related to changes in the regulation of the cholinergic efferent system and in cochlear amplification, which ultimately reverse upon protective hearing suppression. In addition to well-known circuits of the brainstem, the descending corticofugal pathway also regulates efferent neurons of the olivary complex. In this study, we applied our recently developed experimental paradigm of multiple sessions of electrical stimulation (ES) to activate the efferent system in combination with noise overstimulation. ABR thresholds increased 1 and 2 days after AT (8–16 kHz bandpass noise at 107 dB for 90 min) recovering at AT + 14 days. However, after multiple sessions of epidural anodal stimulation, no changes in thresholds were observed following AT. Although an inflammatory response was also observed 1 day after AT in both groups, the counts of reactive macrophages in both experimental conditions suggest decreased inflammation in the epidural stimulation group. Quantitative immunocytochemistry for choline acetyltransferase (ChAT) showed a significant decrease in the size and optical density of the efferent terminals 1 day after AT and a rebound at 14 days, suggesting depletion of the terminals followed by a long-term compensatory response. Such a synthesis recovery was significantly higher upon cortical stimulation. No significant correlation was found between ChAT optical density and size of the buttons in sham controls (SC) and ES/AT + 1day animals; however, significant negative correlations were shown in all other experimental conditions. Therefore, our comparative analysis suggests that cochleotopic cholinergic neurotransmission is also better preserved after multisession epidural stimulation.

## Introduction

The medial olivocochlear (MOC) efferent system enhances hearing sound detection throughout cochlear amplifier regulation (Guinan, 2006, 2010; Lopez-Poveda, 2018), in addition to inducing hearing suppression, as shown in the seminal study by Robert Galambos (Galambos reflex) (Galambos, 1956). More recently, compound action potential (CAP) amplitude suppression and cochlear microphonic (CM) amplitude increments have been shown when applying electrical stimulation on the floor of the IV ventricle (Elgueda et al., 2011). In line with its role in regulating hearing sensitivity, efferent system activation induces a protective effect against noise overstimulation (Handrock and Zeisberg, 1982; Patuzzi and Thompson, 1991; Zheng et al., 1997; Tong et al., 2013; Dinh et al., 2015; Boero et al., 2018). Furthermore, after showing an increased resistance against hearing loss in knock-in mice (KI; Chrna9L9′TKI, carrying a positive alpha 9-receptor point mutation), it has been suggested that MOC cholinergic neurotransmission is directly involved in minimizing noise trauma (Boero et al., 2018).

Electrophysiological evidence also shows that, despite the mechanism of self-regulation of the Galambos’ reflex in the low auditory pathway, the brain cortex also controls efferent olivocochlear (OC) responses (Xiao and Suga, 2002; Terreros and Delano, 2015). Accordingly, descending corticofugal regulation of the strength of the OC reflex has been demonstrated after pharmacological blocking, cooling, or macrostimulation of the auditory cortex (AC) in animal models (León et al., 2012; Dragicevic et al., 2015; Terreros and Delano, 2015). The same effect is detected in humans after cortical epidural electrical stimulation (Fenoy et al., 2006; Perrot et al., 2006).

Short periods of noise overexposure produce reversible changes in hearing loss, known as temporary threshold shifts (TTS). In recent years, the full reversibility of TTS has been questioned after showing that long-term damage of synaptic buttons and afferent fibers persist in overstimulated animals which recover their hearing threshold (Kujawa and Liberman, 2009). These masked alterations, currently known as hidden hearing loss (HHL), can evolve into auditory alterations such as hyperacusis, tinnitus, or difficulties in sound discrimination (Liberman et al., 2016). Thus, short acoustic overexposure with reversible threshold shifts stands out as an overlooked silent alteration, increasingly prevalent in our noisy world, which lacks treatment or prevention (Delano et al., 2020).

In our previous studies, we have recently communicated that chronic anodal epidural stimulation in rats promotes AC activation with transient hearing threshold elevation, as demonstrated by auditory brainstem recordings (ABRs) (Colmenárez-Raga et al., 2019). Based on these results, we hypothesized that a multisession stimulation protocol of the AC may induce a sustained and reversible decrease in hearing sensitivity. Such an effect, also explored in this study, may be used as a potential protective intervention in hearing disorders, such as acoustic trauma or hyperacusis.

Here, we assess the effects on the inner ear of chronic epidural stimulation of the AC in an animal model of transient sound overexposure. More specifically, we analyze the effects of sound overactivation in combination with multisession AC epidural activation in the inner ear of the rat. For this purpose, we applied our previously tested protocol of AC anodal epidural activation (Colmenárez-Raga et al., 2019), followed by a sound stimulation protocol designed for TTS induction [through a single session of 107 dB at a restricted frequency band (8–16 kHz)]. In our experimental approach, the protocol for acoustic stimulation was quite similar to those previously applied by other authors for TTS induction (Kujawa and Liberman, 2009, in mice or Lobarinas et al., 2017, in rats). Ultimately, this study aims to correlate, in a TTS model, the effects on hearing sensitivity of multisession epidural anodal stimulation on the AC (measured by ABR recordings) with MOC efferent cholinergic neurotransmission in the cochlea, analyzed by quantitative immunocytochemistry of choline acetyltransferase (ChAT) in surface preparations of the organ of Corti.

## Materials and Methods

**FIGURE 1:**
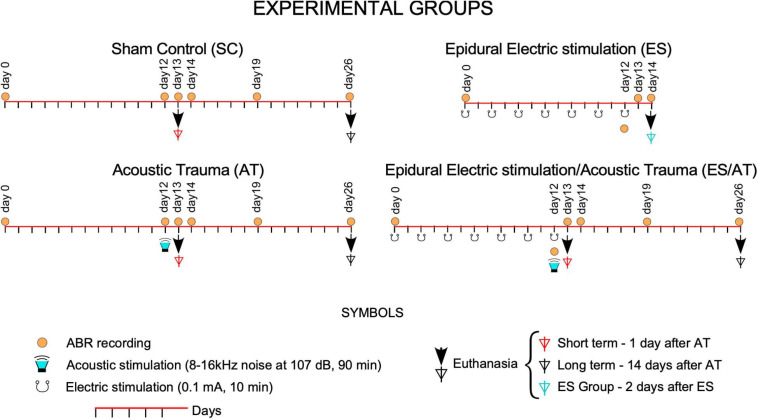
Experimental groups: Timeline of intervention protocols. Over the line of time (red lines), day 0 labels the start of the protocols at 7 days after surgery. From day 0, AT and ES/AT groups underwent acoustic overstimulation at day 12 (green loudspeakers). In groups with electrical stimulation (ES and ES/AT), the multisession protocol was applied from day 0 to day 12. Symbols of ABR recordings (orange circles) and euthanasia (arrows and triangles) were over the curve of time (line in red) following the sequence of events in the protocols.

This study was conducted in strict accordance with Spanish regulations (Royal Decree 53/2013—Law 32/2007) and European Union guidelines (Directive 2010/63/EU) on the care and use of animals in biomedical research. All surgeries were performed under monitored anesthesia (respiratory rate, body temperature, and oxygen saturation), and all efforts were made to minimize suffering. In total, 28 young male Wistar rats weighing from 250 to 300 g, with normal ABR hearing thresholds, were separated into four groups and treated using the following protocols: electrode implantation without any stimulation (electrical or acoustical) (Sham controls, SC), electrically stimulated (ES), acoustic trauma (AT), and electrically stimulated followed by AT (ES/AT) (Figure 1). Furthermore, we assessed the short-term effects of these protocols 1 day after acoustic stimulation (day 13th of the protocol) and the corresponding long-term effects 14 days after AT (day 26th). As shown in Figure 1, the animal groups were organized as follows:

SC—Sham controls (*n* = 12).

ES—Electrical stimulation. Euthanized at day 14th of the protocol (*n* = 3). One of the cases of this group was dropped from further analysis due to damage detected in the deep layers of the AC.

AT—Acoustic trauma. Euthanized at short term (AT + 1 day, day 13th of the protocol) (*n* = 3) and long term (AT + 14 days, day 26th of the protocol) (*n* = 3).

ES/AT—Electrical stimulation and acoustic trauma. Euthanized at short term (ES/AT + 1 day, day 13th of the protocol) (*n* = 3), long term (ES/AT + 14 days, day 26th of the protocol) (*n* = 3).

Sham control rats were histologically processed simultaneously with the treated animals (paired processing of brain sections and inner ear surface preparations).

### Surgery

Under gas anesthesia (2.5% isoflurane), rats were placed in a stereotaxic frame, surgically exposing the left temporal cranial surface. Following the Paxinos and Watson stereotaxic coordinates (Paxinos and Watson, 2005), four points were drawn on the surface of the bone delimiting the borders of the auditory area (for details, see Lamas et al., 2017). An approximately square window was carefully drilled on the bone surface until exposure of the surface of the dura mater. Cold saline (4°C) was dripped to avoid thermal cortical lesions. A 2.25 mm^2^ silver electrode (anode) was gently encrusted into the trepans, and two screws (cathode) were implanted in the contralateral rostral-most side of the skull. After appropriately connecting the system, the electrodes and screws were fully covered by dental cement before any further intervention.

### AC Epidural Stimulation

A 0.1-mA continuous current was delivered for 10 min per session through the epidural bone-attached electrode (anode) using an ISU 200 BIP isolation unit controlled by a CS-20 stimulator (Cibertec, Madrid, Spain). The stability of the voltage current was monitored along sessions. The electrical stimulation protocol was applied in awake animals for seven sessions on alternating days (days 0–12 of the protocol) (Figure 1). For more details, please see Colmenárez-Raga et al. (2019). To assess if the cortical damage after ES enables AC to drive corticofugal responses, serial sections of brain AC were immunostained for GAD 67 in rats from the ES group (please see below).

### ABR Recordings

Recordings were performed under gas anesthesia using a real-time signal processing system [RZ6 Multi I/O Processor, Tucker-Davis Technologies (TDT), Alachua, Fl, United States]. The sound system outputs were calibrated before the recordings using a one-quarter-inch microphone (Brüel and Kjaer). Sound stimuli were 0.1-ms alternating polarity clicks, with a repetition rate of 21 clicks/s delivered in 10-dB ascending steps from 10 to 90 dB. The stimulation sessions were performed in an acoustically isolated chamber. The stimuli were delivered in a close field using a magnetic speaker (MF1 Multi-Field Magnetic Speaker TDT) connected to the ear through a 10-cm-long plastic tube. This approach resulted in a total delay of 1.4 ms in stimulus arrival at the tympanic membrane. ABRs were recorded by averaging 1000 EEG responses to 1000 click stimuli. Three subcutaneous needle electrodes were placed at the vertex and the two mastoids. Evoked potentials were amplified and digitized using a Medusa RA16PA preamplifier and a RA4LI head stage (TDT). Monaural ABRs were recorded from the vertex using the electrode on the mastoid ipsilateral to the click-stimulated ear, as the reference electrode. The needle in the mastoid contralateral to the stimulated ear served as the ground electrode. Monaural ABRs were sequentially recorded by click stimulation in the left and right ears. The placement of the recording electrodes was changed accordingly to record the signals from the side of the sound-stimulated ear. ABR recordings of both sides were analyzed separately. The final signal was filtered with a 500-Hz high-pass filter and a 3,000-Hz low-pass filter (for more details on the ABR recording method, see Colmenárez-Raga et al., 2019). Wave II was first recorded in ABRs and then used to calculate thresholds (©MatLab R-2017 a). The ABR threshold was defined as the minimal sound intensity that evoked a significant voltage change (in a latency range between 1.4 and 5 ms) exceeding the mean ± 2 standard deviations of the voltage value of background activity during the first ms of the recording. The absolute wave latency was defined as time, in milliseconds, from the stimulus onset to the positive peak of the wave. The amplitudes of the ABR waveforms were measured as the peak-to-peak amplitude between the preceding negative trough to the subsequent positive peak of a given wave. In AT and ES/AT groups, ABRs were recorded before and after surgery, as well as 7 days after (day 0), right before AT (day 12) and 1 day (day 13), 2 days (day 14), 7 days (day 19), and 14 days (day 26) after AT (Figure 1).

### Sound Stimulation for Acoustic Trauma (AT)

Awake rats were in a non-reverberant cage with non-parallel sides and exposed to a bandpass noise (8–16 kHz) of 107 dB for 90 min. Noise stimuli were generated digitally (RP2.1, TDT), filtered (RPVDS software), amplified (Audio Source AMP ONE/A), and calibrated inside the cage before each experiment using a one-half-inch microphone (Bruel and Kjäel Instruments, 4134) and a sound level meter with a fast Fourier transform (FFT) analyzer (Larson Davis 831).

### Histology

Animals were deeply anesthetized with an intraperitoneal injection of 6% sodium pentobarbital (60 mg/kg BW) and perfused transcardially with 4% p-formaldehyde in a 0.1-M phosphate buffer (PB). Immediately, cochleae were perfused through the round window, dissected, postfixed for 2 h at room temperature, and decalcified in 8% EDTA for 12 days. Surface preparation membranes were extracted and then dissected into six pieces for whole-mounting processing of the cochlear epithelium. Immunostaining started with a blocking buffer (PBS with 5% normal horse serum and 0.3% Triton X-100) for 3 h, at room temperature, followed by a 2-day incubation at 37°C with the primary antibody, Goat Anti-Choline Acetyltransferase polyclonal antibody (AB144P; Merck Millipore, Temecula, CA, United States) at 1:100. After washing three times in TBS-Tx for 15 min, the dissected pieces were incubated with an anti-goat biotinylated secondary antibody (biotinylated anti-goat IgG H + L, BA-5000; Vector, Burlingame, CA, United States) at 1:200 for 24 h at room temperature. The pieces were then washed with TBS-Tx and incubated for 24 h in avidin/biotin–peroxidase (ABC complex, Vectastain Standard ABC Kit PK-4000; Vector, Burlingame, CA, United States) and further washed with TBS-Tx, followed by Tris–HCl, pH 8.0. They were then incubated in 3,3-diaminobenzidine tetrahydrochloride (DAB; D-9015; Sigma-Aldrich, St. Louis, MO, United States) with 0.006% H_2_O_2_ to visualize the peroxidase reaction. The pieces were finally dehydrated in graded alcohol solutions from 50 to 100%, followed by clearing in xylene and coverslipping.

**FIGURE 2:**
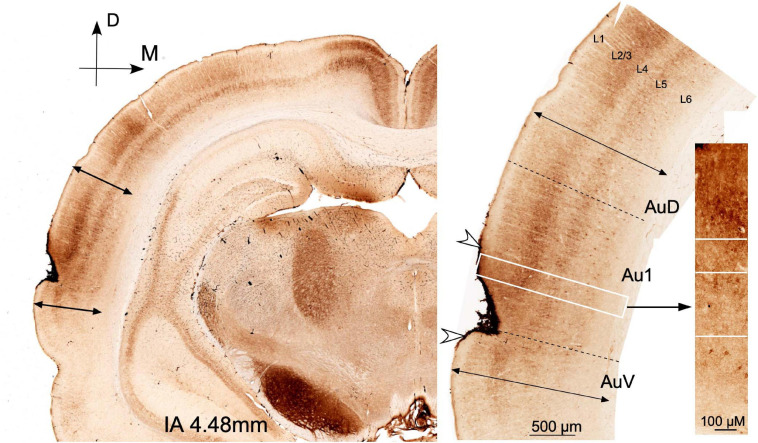
Coronal sections at interaural 4.48 mm (Paxinos atlas coordinates) from a case from group ES euthanized at day 14 of the protocol (2 days after multisession epidural stimulation). GAD 67 dense reaction product in the surface of the cortex defines the position of the electrode. Arrowheads indicate the limits of the lesion in the coronal plane. The perimeter length of the reinforced area in the brain surface was 2.03 mm. Cytoarchitectural landmarks (double arrows) delimit the auditory cortex area. Note the well-defined immunoreactive layers and the well-preserved cytoarchitectural subdivisions in the non-damaged auditory area.

To locate the area of stimulation in the cortex, brains were serially sectioned in the coronal plane into 40-μm sections and immunostained for IBA1 and GFAP, according to our previously published method (Colmenárez-Raga et al., 2019). Glial reaction, both for IBA-1 and GFAP, was delimited on the auditory cortices (data not shown) as previously described by our group. To analyze the state of preservation of the temporal auditory area, after multisession protocol, alternate serial sections of brains from the ES group were stained for Nissl and for GAD 67 monoclonal mouse antibody (Merck Millipore #MAB5406 clone 1G10.2 RRID: AB_2278725) diluted at 1:1,000 TBS 0.05 M + Triton-Tx 0.3% according to the protocol previously described in Pernia et al. (2020) (Figure 2).

### Morphometry and Densitometry

**FIGURE 3:**
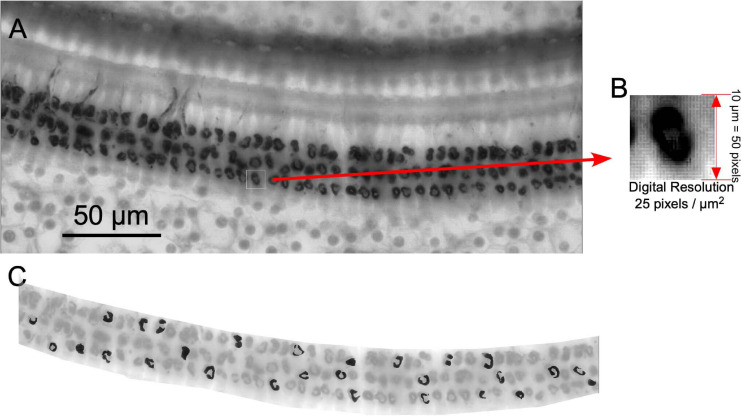
Methodology used for densitometric analysis of immunoreactive buttons. **(A)** Example of a × 40 deep focus picture. **(B)** Digital resolution. **(C)** Thirty terminals per frame (highlighted in black) were manually taken by using freehand tool (ImageJ software).

For cochlear reconstruction, dissected immunostained segments of the organ of Corti (surface preparations) were photographed at × 5 objective and digitized using the Neurolucida software (NL-Vs 8.0, MicroBrightField^®^, Inc., Williston, VT, United States) under a Leica DMRX microscope equipped with a set of plan apochromatic objectives. Pictures from each slide were combined and ordered cochleotopically using as reference changes in the width of the organ of Corti and the thickness and density of the spiral bundle. Using this approach, a single final image of the whole cochlea was recomposed using the Canvas software (Canvas Draw 5 for Mac). After digital reconstruction, a line was drawn along the spiral bundle (SB) to calculate the cochlear length. These lines were measured using the Canvas perimeter tool. The length of the rat basilar membrane has been previously analyzed and estimated as 9.4 mm for Wistar Rats (Burda et al., 1988). In our samples, the mean perimeter of all cochleae measured was 8.08 mm (SD 0.79). According to previous measurements (Burda et al., 1988), our larger cochlear reconstruction was 9.4 mm in length. By using the “measure line” plugin of the ImageJ software program, provided by Eaton-Peabody Laboratories, the locations of several frequencies in the reconstructed cochlea were labeled for subsequent topographic cochleotopic analysis (see below). Once the frequencies were located in the cochlea, six pictures per cochlea (×40 objective) centered on 2.8, 8, 11.3, 16, 32, and 45.2 kHz were captured using the deep focus tool from Neurolucida 8.0 (MBF Bioscience, Williston, Vermont, United States). To obtain the resulting deep-focus image, five 1-μm images were Z-stacked using the first surface plane of sharp focus of immunoreactive terminals as a reference. The acquired images were processed for morphometric and densitometric analysis of ChAT immunostained buttons using the software ImageJ. Both densitometric and morphometry analyses were performed after separating (digital cutting) the entire OHC area in the pictures with the free hand tool of the ImageJ software program (Figure 3).

### Morphometry

#### Size and Number of Terminals

Images (×40) with a digital resolution of 25 pixels/μm^2^ were used for morphometry (Figure 3A). For segmentation of ChAT-ir cell buttons, thresholding operations were further applied with ImageJ. Thresholding was applied to all images, followed by automatic counting of all selected particles. Terminal immunoreactive buttons were segmented using density thresholding in the ImageJ software program. The number of segmented buttons was normalized to N/10,000 μm^2^ surface area.

### Densitometry

Before capturing, the illumination source of the microscope was adjusted using a stepped density filter (11 levels) (^®^EO Edmund industrial optics—ref 32599, Karlsruhe, Germany). In total, 30 buttons per ×40 image (equivalent to one frequency sample) were manually segmented using the ImageJ freehand selection tool (Figure 3). The density values of immunoreactive terminals were determined using the ImageJ software. The mean gray level of the neurons (a value between 0 and 255) was used as a measure of the button immunoreactivity to ChAT. We used the values of microscopic illumination determined using the density step filters (see above) to translate gray values into optical density (OD) values. In this paper, normalized gray OD levels were used instead of direct gray-level measures. The normalized gray levels were calculated by subtracting the mean of OD of the field (value of the entire OHC region) from the OD level of the immunoreactive buttons and by dividing the result by the standard deviation of the entire field.

### Statistical Analysis

Statistical analysis was performed using the IBM^®^ SPSS^®^ software, version 25 (IBM Corp. and SPSS Inc., Chicago, IL, United States, RRID: SCR_002865). Differences in ABR thresholds values between different record times within each group were analyzed using the non-parametric Friedman test followed by Bonferroni *post hoc*. Comparisons between groups at each recording time were performed using the Mann–Whitney test. No significant differences were found when comparing recordings of the left and right ears of SC and stimulated animals.

For quantitative immunocytochemistry, one-way ANOVA followed by the Bonferroni and Games–Howell *post hoc* tests were used to assess differences between groups in OD, number/10,000 μm^2^, and size of ChAT immunoreactive terminal buttons. Differences between groups by frequencies were assessed by two-way ANOVA. Spearman’s rank and Pearson correlation coefficients were used to analyze correlations between size and OD measurements of immunoreactive terminals. Differences were considered significant at *p* < 0.05.

## Results

### ABR Recordings

**FIGURE 4:**
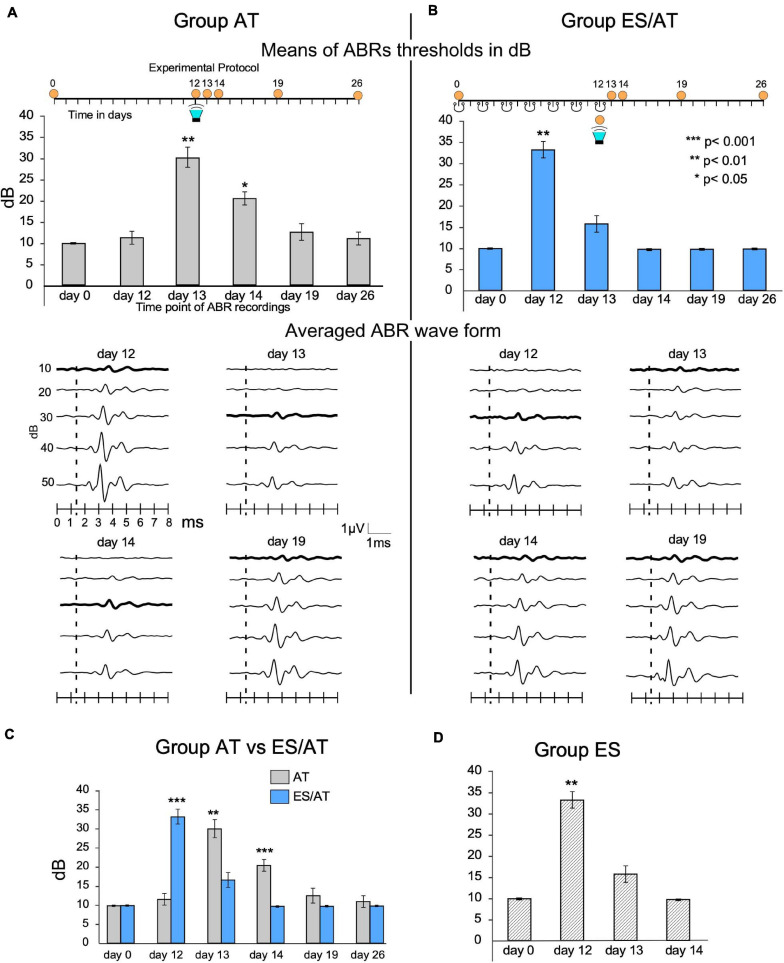
Thresholds and waveform analysis of ABRs. **(A)** The AT group shows a significant threshold shift at AT + 1 day (day 13) and AT + 2 days (day 14). **(B)** The ES/AT group shows a significant threshold shift before, but not after, overstimulation (the green loudspeaker labels acoustic stimulation). Waveform analysis is shown in the middle of the panel. Lines represent averaged waves from all animals of each group. The thickest lines label averaged thresholds. **(C)** Statistical comparison of the mean thresholds of groups AT vs. ES/AT. Note significant differences in threshold shifts after AT at day 13, the day after acoustic stimulation. Please also note that, in the days after the recordings, the thresholds recover later in the AT group than in the ES/AT group. **(D)** ES group. Multisession epidural stimulation induces threshold shifts immediately after the last stimulation on day 12, which recovers 1 day later (day 13).

SC animals showed regular, constant 10-dB ABR thresholds in recordings at different timepoints of the protocol. In the AT group, the thresholds significantly increased at AT + 1 day (day 13) (30 ± 6.32 dB, *p* < 0.01) and AT + 2 days (day 14) (21.66 ± 4.08 dB, *p* < 0.05) (Figure 4A) and decreased at AT + 7 days (day 19) (13.33 ± 5.16 dB), albeit non-significantly, until reaching values similar to those of pretreated rats at AT + 14 days (day 26) (Figure 4A). In the ES/AT group, during the pretreatment period (before acoustic stimulation and after surgery, from 0 to 12 days), the animals received seven sessions of epidural electrical stimulation on alternating days (Figure 4B). After this sequence of cortical stimulation, and before AT, the mean thresholds increased to 33.33 dB (SD 5.16) (Figure 4B). However, no significant differences in mean thresholds were found at AT + 1 day (day 13) (Figure 4B). The comparison of the thresholds at the same stages of the protocol AT + 1 day (day 13) and AT + 2 days (day 14) between the AT and the ES/AT groups shows that the means of the AT group are significantly higher than those of the ES/AT group (Figure 4C). In the ES group, the thresholds increased after the last session of epidural stimulation (day 12), as shown in the ABR recordings. The mean threshold values reached normal levels at ES + 1 day (day 13) (Figure 4D).

### Brain Cortex Preservation After Multisession Stimulation

The localization and extension of the electrical stimulated area in the brain cortex was analyzed using glial immunocytochemical markers (GFAP and IBA-1) and Nissl staining in alternate sections, following the approach previously applied by our group (Colmenárez-Raga et al., 2019). In addition, to test the state of preservation of the cortical microcircuitry in the ES animal group, serial sections were stained for GAD 67 (Figure 2). As observed in our previous study, all areas of glial reaction highlighted by glial markers were restricted to the auditory temporal area. Furthermore, the more superficial layers were affected in varying degrees, depending on how the electrode is encrusted into the skull [data not shown; please refer to Colmenárez-Raga et al. (2019) for further details].

Denser GAD 67 immunoreactivity in the dura and superficial layers of the cortex makes it possible to define the extension of the damaged areas (Figure 2). Immunoreactive GAD neurons and terminal fields are present virtually throughout the auditory cortices despite a ribbon under the dura (Figure 2). Around the area of contact of the electrode, the cytoarchitecture and layering of the auditory temporal area can be easily differentiated through cases, thus indicating that cortical microcircuits beyond the damaged region are well preserved (Figure 2).

### Anatomy

#### General Features From Sham Controls

**FIGURE 5:**
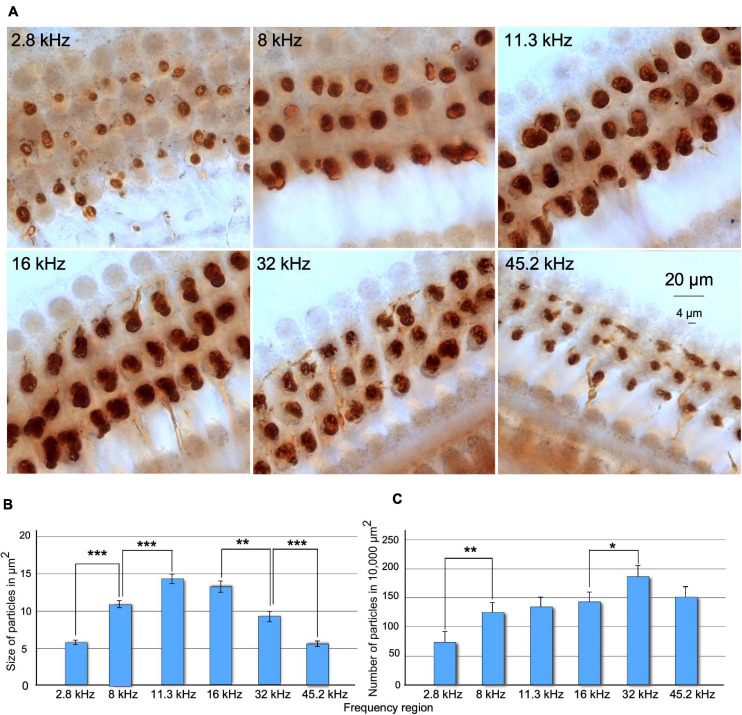
**(A)** Comparison of changes in size and number of ChAT-immunostained terminal buttons along frequency regions of the cochlea in SCs. Images were acquired using a deep focus tool. **(B)** Statistical analysis of size of buttons upon density threshold segmentation. Note that the size of the terminals increases and decreases along the tonotopic axis of the cochlea. Significant differences were found when comparing each frequency region with the adjacent, except between 11.3 and 16 kHz regions. **(C)** Number of terminals measured by density threshold segmentation normalized to 10,000 μm^2^. The lower significant number of the terminals was found at 2.8-kHz areas and the highest at 32-kHz areas. After comparing adjacent frequency regions, significant differences were found between 2.8 and 8 kHz and 16 and 45 kHz (*p > 0.05, **p < 0.01, ***p < 0.001).

Under the microscope, immunostained preparations showed thick, myelinated fascicles of positive efferent fibers across the spiral limbus of Huschke and along the floor of the tunnel of Corti. Fascicles penetrated in, ramified at, and meandered around the deeper section of supporting phalangeal cells. After a short ascent, the fibers ended in terminals and innervated the basal pole of the OHC. A dense dark-brown reaction product sharply defined the size and shape of efferent terminals along the cochlea (not shown). Differences in size, density, and number of immunoreactive buttons were observed along the frequency range (Figure 5A). When comparing buttons between cochleotopic regions, the higher the frequency, the more regular the distribution in rows and the shape of immunoreactive buttons would be (Figure 5A). Values in the size of the buttons increased gradually in mid-frequency regions and decreased in high-frequency regions (Figure 5B). Overall, normalized numbers of segmented particles indicate a gradual increase, along the cochlear axis, from low- to high-frequency regions (Figure 5C).

### Inflammatory Response

**FIGURE 6:**
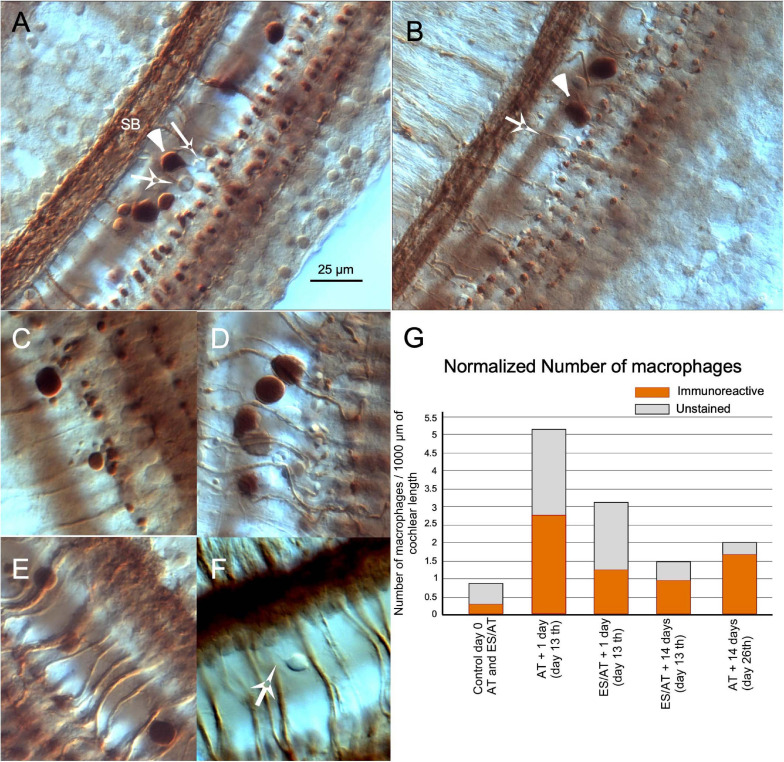
Inflammatory reaction in the cochlea after acoustic trauma. **(A)** Panoramic view of the organ of Corti in surface preparation. Large immunoreactive inflammatory cells (arrowheads) are interspersed with other smaller non-immunoreactive (arrows). **(B)** Inflammatory cells, presumably macrophages, migrating to the OHC area. **(C,D)** Details of close contacts of immune cells with immunoreactive terminals. Cell debris is associated with the loss of terminals. **(E)** Immune cells climbing along efferent fibers. **(F)** Presumably monocytes with filopodia, most frequently observed in the floor of the tunnel. Differential interference contrast microscopy (Nomarski) (×63 PLAN APO Leica/NA 1.32-0.6 immersion oil). **(G)** Quantification of the immune response by counting separately the number of immunoreactive and unstained macrophages. The number of inflammatory cells is higher after AT in the AT group and decreases at 14 days (day 26th). SB spiral bundle.

In both SCs and stimulated cochlea (AT and ES/AT), free cells were more frequently found in the tunnel of Corti. These cells were irregular in shape and variable in size (from 5 to 20 μm) (Figure 6). The largest cells (10–20 μm), which showed intense ChAT immunoreactivity, were spherical and contained filopodia and pseudopodia, features which identify them as macrophages (Figure 6 arrowheads). The smallest cells (about 5–10 μm) were not immunopositive but also had filopodia and were thus compatible with monocytes (Figures 6A,B,F white arrows). A few of these cells were found in control cochleae. For this reason, we considered them as a casual finding at the beginning of the microscopic observation. Some of these free cells have been found in our material associated with the loss of efferent synapses and the presence of debris, which suggest an active digestion of terminal buttons (Figures 6B–D). In surface preparations of stimulated cases (AT and ES/AT), the number of macrophages was extremely variable with a random distribution along the cochlea. After separately counting immunoreactive and non-immunoreactive cells and normalizing the values (per 1,000 μm of cochlear length), a higher number of cells were tallied AT + 1 day (day 13) than ES/AT + 1 day (Figure 6G). In both experimental conditions (AT and ES/AT), the values of number of reactive and non-reactive inflammatory cells decreased at AT + 14 days (day 26) (Figure 6G).

### Cholinergic Olivocochlear Terminal Buttons. Quantitative Immunocytochemistry

### Morphometry

**FIGURE 7:**
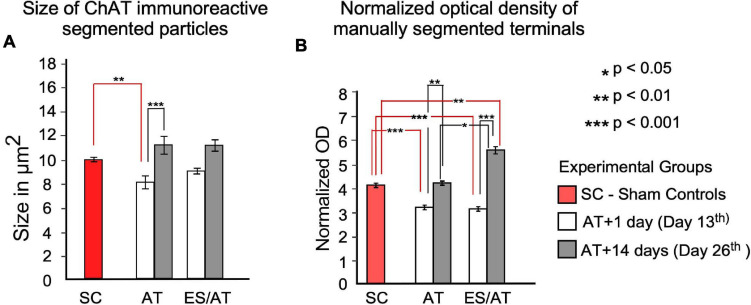
Area and OD statistical analysis of ChAT-immunoreactive terminals through the cochlea. **(A)** Size of the particles. Differences were significant between the SC group and AT + 1 day (day 13) (red lines indicate this comparison). After comparing day 1 (day 13) and 14 (day 26) days after overstimulation, significant differences were identified only in the AT group (black lines). No significant differences were found after any comparison in the ES/AT group. **(B)** OD analysis of efferent terminals. Values are significantly lower at 1 day after overstimulation (day 13) under both experimental conditions (with or without ES). A rebound in values, significantly higher in the ES/AT group, was found at 14 days after overstimulation (day 26). *p < 0.05, **p < 0.01, ***p < 0.001.

After counting immunoreactive buttons by density threshold segmentation, a statistically significant loss of terminals was not detected in any experimental groups when comparing with SC (data not shown). A significant decrease in the size of terminals was detected only in group AT + 1 day (day 13) when analyzing the cochlea as a whole (*F* = 3.639; *p* < 0.01) (Figure 7A). However, in both experimental groups (AT and ES/AT) a non-significant decrease was also observed in ES/AT + 1 day (day 13), with a recovery of the values at 14 days after AT (day 26) (Figure 7A).

### Densitometry

Analysis of the whole cochlea showed a significant decrease in normalized OD values when comparing SC with both experimental conditions (AT and ES/AT) at 1 day after acoustic trauma (day 13) (*F* = 3.548; *p* < 0.001) (Figure 7B). In addition, OD values significantly rebounded in both experimental conditions (AT or ES/AT) at 14 days after acoustic trauma (day 26), although the ES/AT values were significantly higher (Figure 7B).

### Analysis by Frequency Regions

**FIGURE 8:**
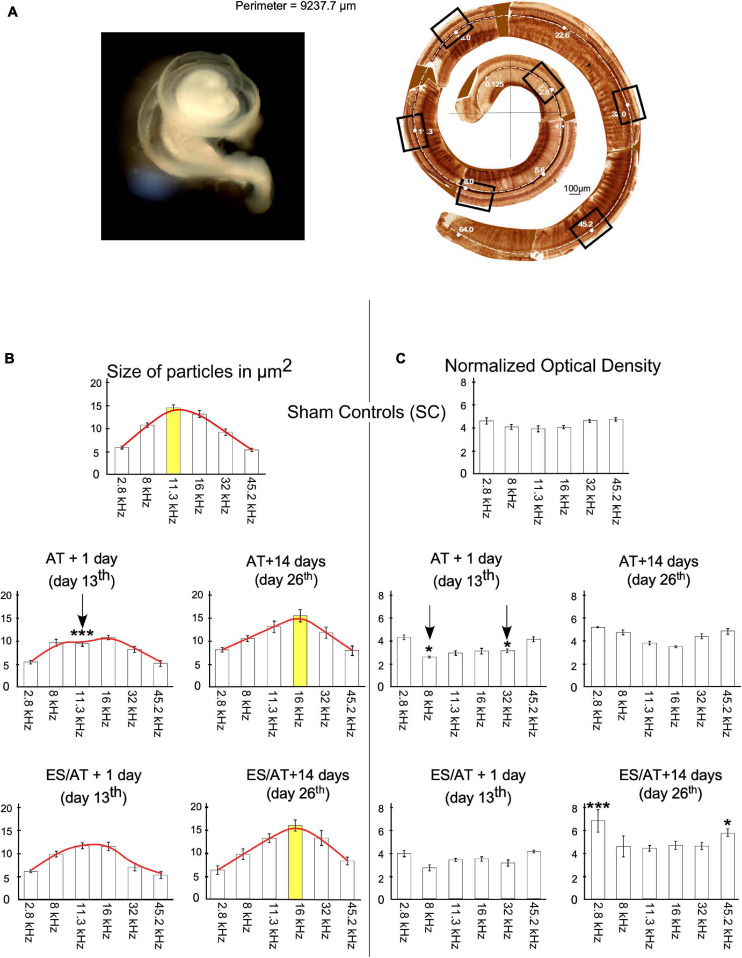
**(A)** Image on the right shows membranous labyrinth of the cochlea after dissection. On the right, cochlear reconstruction was performed using digitally glued images from immunostained Organ of Corti surface preparation. Total length of the cochlea in this example was 9.237.7 μm, which ensures a complete extraction and accurately allows to define frequency regions. Squares delimit frames of the photographs taken to analyze a significant sample of six frequency regions. **(B)** Quantitative analysis of size by frequency region. Each bar in the graphs corresponds to one frequency region. Red lines on the top of the bars highlight the evolution of means of size values along cochleotopic regions. Note the changes in shape of the enveloping red lines in relation to SC at 14 days (day 26). Asterisks indicate significant differences between the experimental groups and SC for each frequency. Yellow bars indicate frequencies of higher values in SC, AT, and ES/AT groups at 14 days after AT (day 26). Arrows indicate significant decreases in values in the 11.3-kHz region at AT + 1 day (day 13). **(C)** Significant decrease in OD values at AT + 1 day (day 13) in the 8- and 32-kHz regions and increases in 2.8 and 45.2 kHz, in the ES/AT group.

Six frequency regions were selected along the tonotopical axis per cochlea (see “Materials and Methods” section) and analyzed to assess changes in size and OD of immunoreactive terminals (squares in cochlear reconstruction in Figure 8A). In the SC group, the values of the size of buttons gradually increase and then decrease across frequencies, showing a Gaussian distribution, peaking at 11.3 kHz (Figure 8B top). In comparison, the size of terminal buttons in AT and ES/AT animals decreased at AT + 1 day (day 13), mostly in the middle-frequency regions (please see Figure 8B, red lines in AT + 1 day and ES/AT + 1 day). Changes in the shape of the lines connecting mean size values allows a better understanding of the evolution of the parameters along frequency areas (envelopes—red lines in Figure 8B). Statistical comparison of the values for each frequency region with the SC group showed a significant decrease in the 11.3-kHz region at AT + 1 day (day 13) (Figure 8B, arrow). In addition, at 14 days after ES (day 26), the size for terminals increases again for the middle frequencies in both groups (AT and ES/AT) recovering the Gaussian distribution observed in SC (Figure 8B right). Interestingly, the largest terminals appear now in the 16-kHz region, in contrast to peak in the 11.3-kHz region of the SCs (Figure 8B—compare bars highlighted in yellow).

The distribution of OD values in the SC group showed no significant differences across frequency regions (Figure 8C—top). However, a significant decrease in values was evident in AT + 1 day (day 13), in the 8- and 32-kHz regions (Figure 8C—arrows). Such decrement was not found in the ES/AT group (Figure 8C bottom). On the other hand, at day 26 of the protocol, the OD is increased compared to the sham controls in group ES/AT in the 2.8- and 45.2-kHz regions (Figure 8C bottom). After correlation test analysis of both parameters (size and OD), all experimental groups, except ES/AT + 1 day (day 13), showed a significant correlation (Table 1). We suggest that this finding speaks in favor of a better-preserved cochleotopy (more similar from controls) 1 day after ES.

**TABLE 1:** Correlation test.

Animal groups	Means/SD	Means/SD	Coefficient	*p*-value
	of OD	of area		
Control	4.30/1.25	10.00/4.63	−0.123	*p* > 0.05
AT + 1 day	3.37/0.84	8.14/2.51	−0.584	*p* < 0,001
EE/AT + 1 day	3,26/0,79	9,11/3,11	−0.269	*p* > 0,05
AT + 14 days	4,34/0,69	11,36/3,73	−0.578	*p* < 0.001
EE/AT + 14 days	5.14/1.90	11.33/4.45	−0.391	*p* < 0.001

## Discussion

In this study, we have shown that multiple sessions of electrical activation of the AC before sound overstimulation preserve hearing thresholds and curtail the inflammatory response in the cochlea without a significant loss of terminals. Furthermore, sound overstimulation significantly reduces the size of the immunoreactive cholinergic buttons 1 day after acoustic overstimulation (day 13) in the AT group, but not after cortical electric activation in the ES/AT group as shown by ChAT quantitative immunocytochemistry in cochlear surface preparations. The OD values of the ChAT immunoreaction products decrease in both experimental groups (AT and ES/AT) at 1 day after sound overstimulation (day 13). The values of both parameters (size and OD) recover at 14 days after acoustic overstimulation (day 26) despite OD means being significantly higher in ES/AT than in AT.

The analysis of the normalized measurements of immunoreactive buttons by frequency region shows statistically significant decreases only in AT + 1 day (day 13), for both size (at 11.3 kHz) and OD (at 8 and 32 kHz). Correlation test analysis for both parameters (size and OD) shows no significance only in group ES/AT + 1 day (day 13).

### Animal Model

#### Cortical Effects of Epidural Stimulation

Changes in GAD immunoreactivity after cortical damage were previously analyzed by our group in a model of restricted ablation of the AC, showing that this marker for GABA neurons allows to define the limits of the lesion as well as the cortical cytoarchitectural subdivisions (Lamas et al., 2013). Inhibitory microcircuits (GAD-GABA) are crucial for neuronal network regulation (Kawaguchi, 2017) and indirectly reflect, if well preserved, potential effectiveness for driving responses of the brain cortex. Both present results (Figure 2) and our previous analysis of the effects of cortical multisession electric stimulation (Colmenárez-Raga et al., 2019) have shown that injuries in the cortex after electrode activation are restricted to relatively small areas of the auditory temporal area. Moreover, descending pathway activation of ACs can be ensured in our material, since the deeper cortical layers (layers 5 and 6, where corticofugal neurons are located) are not significantly affected (Figure 2). The size and shape of the damaged temporal cortex, as shown in reconstructions from our previous publication (please see Figure 9 in Colmenárez-Raga et al., 2019) and the well-preserved GAD cytoarchitecture (present results, Figure 2), indicate that auditory cortices remain functional after the protocol of ES. Since stimulation is unilateral in our model, excitatory callosal connections may also contribute to drive the corticofugal neurons of the contralateral side. Although residual plasticity effects cannot be fully ruled out in our experimental approach, marked increases and decreases in ABR threshold shifts also indicate a dynamic active feedback regulation of cortical neuronal networks over time. Notwithstanding anatomical analysis of AC preservation after stimulation, minimal lesions should be considered out of safety limits.

#### Sound Overstimulation

Our experimental approach to overstimulation was quite similar to the one published by Lobarinas et al. (2017). These authors reported (after subjecting rats to 2 h of band-pass noise of 8–16 kHz at 106 dB) threshold shifts ranging from 20 to 25 dB, approximately 1 day after sound overstimulation and a long-term full recovery, as shown in our animal model. Furthermore, in this paper, tonal ABRs show threshold elevations with a linear increase in the values from lower to higher frequencies and with a decrease in distortion product otoacoustic emissions (DPOAE) amplitudes (Lobarinas et al., 2017).

In a similar overstimulation protocol in mice, threshold shifts 1 and 2 days after overstimulation recovered at 8 weeks after applying a band noise of 8–16 kHz (100 dB, for 2 h in free field) (Kujawa and Liberman, 2009). These authors also reported an acute loss of synaptic ribbons in hair cells, which may have functionally silenced neurotransmission despite the complete recovery of hair cell function. Such an alteration, known as hidden hearing loss (HHL), supports an underlying long-term alteration of neurotransmission to spiral ganglion dendrites, after which auditory thresholds resume normal values. However, cochlear nerve responses depend not only on glutamatergic neurotransmission of IHCs but also on efferent cholinergic neurotransmission of outer hair cells, which indeed regulates micromechanically its responses (Malmierca and Merchán, 2004). Our results show changes in efferent neurotransmission with normal thresholds at 14 days after AT (day 26) (Figures 7, 8). Following this line of thinking, we suggest that our protocol, or other similar ones, should be explored in the future at longer survival times together with a combined evaluation of afferent and efferent neurotransmission.

#### Hearing Sensitivity (ABRs)

Multiple sessions of ES before sound overstimulation induce hearing suppression, as shown by elevated thresholds after ABR recordings (Figures 4B,D), thus confirming our previously reported results using a similar stimulation protocol (Colmenárez-Raga et al., 2019). Due to the excitatory character of the corticofugal descending pathway (Feliciano and Potashner, 2002), anodal stimulation of the AC can drive the direct (cortico-olivary) and indirect (*via* inferior colliculus IC) corticofugal pathway (Horváth et al., 2003), which ultimately activates MOC olivary neurons. From an anatomical point of view, direct connections from infragranular layers of the AC and from the IC to the olivary complex support a descending corticofugal activation of VNTB–MOC neurons (Spangler et al., 1987; Vetter et al., 1993; Malmierca et al., 1996; Saldaña et al., 1996; Weedman and Ryugo, 1996; Winer et al., 1998; Schofield and Cant, 1999; Thompson and Schofield, 2000; Senatorov and Hu, 2002; Doucet et al., 2003; Warr and Boche, 2003; Bajo and Moore, 2005; Bajo et al., 2007; Malmierca and Ryugo, 2011; Mellott et al., 2014; Straka et al., 2015). Thus, in our protocol, persistent threshold shifts, shown after the end of cortical electrical stimulation (day 13) (Figures 4B,D), can be explained by a sustained activation of synaptic plasticity machinery in cortical networks. Such cortical activation has also been demonstrated after daily anodal transcranial direct current stimulation, which induces a persistent neural excitation and overexpression of plasticity-associated genes in the sensorimotor cortex (Kim et al., 2017). Moreover, after AC restricted ablation in the rat, the AC is able to trigger plasticity in the organ of Corti, inducing stable and long-term changes in the expression of motor proteins (Prestin and ß Actin), as previously shown by our group (Lamas et al., 2013, 2014). Considering the roles of the efferent system in hearing (Guinan, 2010), threshold elevation, after ES, can be explained by sustained and persistent cortico-olivary activation followed by MOC and/or inner ear plastic responses. Therefore, the effect of ES in decreasing hearing sensitivity (hearing suppression), before sound overstimulation (Figures 4B,D), is the most plausible explanation for differences in changes in threshold shifts over time between the AT and the ES/AT experimental groups. In addition, in the ES/AT group, the threshold shift occurred before AT, with normal thresholds at 1 day after AT (day 13); hence, changes in immunoreactivity parameters are most likely primarily related to cortical stimulation, rather than to AT, under this experimental condition, as discussed below. Synaptic plasticity activation has been demonstrated by whole-cell patch-clamp recordings, showing that layer 5 neurons can respond through long-term potentiation (LTP) or long-term depression (LTD), after layer 6 stimulation in AC slices (Kotak et al., 2007). Also, tDCS stimulation increases cortical neuronal metaplasticity in AC neuronal networks (LTP or LTD) (Nitsche et al., 2008; Zhang, 2013). Moreover, after repetitive stimulation of the cortex, neurons develop a sustained increase in firing rate for hours (Nitsche and Paulus, 2000). In our recordings, presumable increases in the excitability and firing rate of corticofugal neurons of layers 5 or 6 after repetitive anodal stimulation can be related to MOC neuron activation. Threshold shifts induced by overstimulation depend primarily on reflex arc activation in the low auditory pathway (De Venecia et al., 2005). However, after electric stimulation of the temporal area and acoustic overstimulation, both feedbacks (reflex arc and cortico-olivary) involved in cochlear amplification work in combination. Therefore, the increase in thresholds after AT may be driven by persistent metaplasticity (LTP) of the epidurally stimulated cortex. Indeed, the recovery of thresholds at ES/AT + 1 day (day 13) indicates a compensation net induced by an overactivated corticofugal pathway acting on MOC olivary neurons.

### Technical Limitations

The click stimulus used to record the ABRs is a broadband stimulus covering a wide range of low frequencies (<10 kHz). Although this click stimulus can be used to successfully measure threshold shifts in rats (see Figure 4), it may not show the potential contribution of high frequencies to transient threshold shifts. Accordingly, future studies using tonal ABRs may shed light on putative differences in threshold at low and high frequencies.

### Quantitative Immunocytochemistry

ACh is synthetized in the soma and terminals of the neurons by the combined choline and acetyl CoA reaction catalyzed by ChAT. ACh is delivered into the synaptic clefts and coupled by receptors, and the remaining neurotransmitter is hydrolyzed by the enzyme acetylcholinesterase (AChE); concurrently, choline reuptake in the terminals enables its coupling with acetyl CoA (Simon and Kuhar, 1976; Kuhar, 1979; Matsuo et al., 2011). Consistently, neurotransmitter storage in terminals depends on the rate of synaptic delivery of ACh, on balanced *de novo* synthesis, and on neurotransmitter recycling. Our measurements of the size and OD of the buttons, which quantitatively indicate the amount of reaction product, and ultimately the rate of ACh synthesis, reflect the state of synaptic neurotransmission. Thus, decreases in the size and OD of efferent terminals shown by us reflect synaptic depletion, after sound overactivation. After sound overstimulation, AChE (the enzyme involved in ACh recycling) immunoreactivity in the organ of Corti decreases in guinea pigs (Mounier-Kuhn and Haguenauer, 1967) and chinchilla (Kokko-Cunningham and Ades, 1976) according with our results. In our samples, the size of the terminals significantly decreased in AT + 1 day (day 13) but not in ES/AT + 1 day (day 13). Thus, the differential values of terminal button quantitative analysis, assessed in ES/AT, can be related to a mitigated noise effect induced by hearing suppression. The significant rebound of OD values in the ES/AT group 14 days after AT (day 26) suggests a recovery in ACh synthesis after depletion by acoustic trauma. However, significant differences in OD values in the ES/AT group at 14 days (day 26) after AT (Figure 8C bottom), which were not observed in the AT + 14 days (day 26) group, may also be related to the activation of long-term cortical plasticity by electrical stimulation. In the ES/AT group at 1 day and 14 days after AT, quantitative analysis shows that changes in values of quantitative immunocytochemistry result in combination with normal thresholds (Figures 4, 7). These results suggest that temporal windows for stabilization of efferent neurotransmission do not match hearing sensitivity recovery. Future analysis of correlation of efferent and afferent system alterations at long term will be needed to shed light on this problem.

### Analysis by Frequency Regions

According to a previous ultrastructural analysis of ChAT immunoreactivity in the organ of Corti, MOC terminals in the OHC are compact and densely filled with reaction products (Eybalin and Pujol, 1987), as shown in our light microscopy images. Consequently, fulfilled terminals with a homogeneous immunoreactive product, shown in our material, ensure that our measurements detect accurately the actual size of the buttons. Synaptic size is affected by multiple molecular mechanisms, some of which depend on dynamic synaptic activation, whereas others remain unaffected (Lund and Lund, 1976; Pierce and Milner, 2001; Stanic et al., 2003; De Paola et al., 2006; Pasantes-Morales and Tuz, 2006; Grillo et al., 2013; Petrof and Sherman, 2013; Statman et al., 2014; Pasaoglu and Schikorski, 2016; Sammons et al., 2018). Cochleotopic analysis of the size of immunoreactive terminals in our control animals shows a progressive increase from low-to-medium-frequency areas and a gradual decrease to high-frequency ones (Figure 8B). The bell distribution of size values in the SC group is replaced by a more homogeneous distribution at AT + 1 day (day 13) and ES/AT + 1 day (day 13) due to the decrease in the size of the buttons at the middle range of frequencies (8–16 kHz). These results suggest a more intense effect of frequencies at the noise band (8–16 kHz) used for sound stimulation (Figure 8B). Unlike the size of the terminals, the OD values, as assessed by frequency region, show a flat distribution in the SC group, with slightly higher values at the ends of the frequency range (Figure 8C). In fact, OD measures the amount of ChAT in synaptic efferent buttons and, indirectly, the rate of ACh synthesis. A significant decrease in OD values of AT + 1 day (day 13) in the frequency regions of 8 and 32 kHz (not shown in the electrical stimulated group) may reflect unrecovered neurotransmitter synthesis in mid-frequency regions after depletion of terminals by overactivation (Figure 8C). Tonotopical analysis of ABRs in rats, applying a similar TTS protocol (107 dB—frequency band of 8–32 kHz—90 min of sound exposure) and with a similar timeline (1 day and 2 weeks), has shown that, although thresholds returned to baseline, wave 1 amplitudes at 16, 24, and 32 kHz failed to return to control levels (Lobarinas et al., 2017). Significant changes in size and OD have been shown in our stimulated groups in similar ranges of frequencies at 1 day post stimulus but not at 14 days (Figures 8B,C); however, recovery was observed at 14 days after exposure. This apparent discrepancy in tonotopic effects of AT between our anatomical results and those of Lobarinas et al. (2017) can be related to a delayed recovery of ACh synthesis with respect to wave-amplitude thresholds. No significant changes in size or OD have been shown in the ES/AT + 1 day (day 13th) group across frequency regions. Conversely, significant differences were shown in both parameters at AT + 1 day (day 13th) (vertical arrows in Figures 8B,C). It seems relevant to remark that all experimental groups, except ES/AT + 1 day (day 13th), showed a significant correlation, after correlation test analysis, which suggests better-preserved cochleotopy (more similar from SC) induced by electric stimulation of the temporal cortex (Table 1).

### Inflammatory Response

Immune responses, primarily involving monocytes and macrophages in the cochlea, have been shown after sound overstimulation (Fredelius and Rask-Andersen, 1990; Hirose et al., 2005; Wood and Zuo, 2017; Frye et al., 2019; He et al., 2020, among others).

The cochlear immune response includes resident cells, which can actuate by humoral liberation of inflammatory mediators (i.e., supporting cells and lateral wall fibrocytes, among others) (Cai et al., 2013) and mobile cells (macrophages). Such cochlear cleaners are located in the basilar membrane, as silent monocytes, which migrate to the sensory epithelium after activation by cochlear damage (Fredelius and Rask-Andersen, 1990; Frye et al., 2017). Our microscopic observations show ChAT-immunoreactive cells, variable in size (5–20 μm in diameter) and shape (irregular or globular), usually with filopodia, mainly located in the tunnel of Corti, which can be anatomically identifiable as macrophages (Figure 6). These cells have been closely related to areas of cell debris and loss of immunoreactive buttons (Figures 6A–D). Collateral pruning by microglial cells is currently considered a physiological mechanism of regulation of network connectivity and plasticity. Accordingly, microglial amputation of buttons occurs in neurological diseases, such as Parkinson’s disease, Alzheimer’s disease (Hong et al., 2016), epilepsy (Andoh et al., 2019), or schizophrenia (Sellgren et al., 2019). Whether or not macrophages, in our animal model, effectively or extensively participate in the remodeling of efferent terminal fields in damaged cochlea remains unknown, but this is undoubtedly an interesting question, which merits further research in the near future.

Considering that some ChAT immunoreactivity was detected interstitially, cochlear macrophagic cells may also be involved in actively removing the enzyme from the perilymph. However, dendritic macrophages physiologically express choline acetyltransferase (ChAT), muscarinic and nicotinic acetylcholine (ACh) receptors, and acetylcholinesterase (AChE) (Fujii et al., 2017). In principle, their potential constitutive molecular profile may also explain their immunoreactivity. Yet, small cells remained unstained, and the immunoreaction was mainly detected in the larger cells. This finding supports the hypothesis that reactive macrophages may act as cleaners of ChAT, after neurotransmitter depletion in the efferent terminals. Our normalized cell counts showed that the number of macrophages is higher in AT + 1 day (day 13) than in ES/AT + 1 day (day 13) (Figure 6G), which suggests that the immunoreaction (and presumably the cochlear damage) is lower after cortical electrical stimulation.

## Concluding Remarks and Clinical Implications

In this paper, we have shown that multisession ES prevents threshold shifts and minimizes inflammatory reaction after acoustic overstimulation in our animal model of TTS. Consequently, auditory temporal area stimulation can be considered a potential approach to hearing preservation after mild sound overstimulation. ChAT quantitative immunocytochemistry results also indicate that TTS induces short-term neurotransmitter depletion of cholinergic terminals apposed on OHC, which recovers in the long term. Significant long-term increases in the amount of neurotransmitter in terminals (OD analysis), in the electrically stimulated experimental group, indicate persistent plastic activation of the MOC with normal thresholds, which should be explored in future research. Furthermore, the widening range of sizes and differences in OD along the cochleotopic axis suggests that chronic multisession anodal stimulation helps also to preserve the tonotopic neurotransmission of the efferent olivocochlear terminals in the inner ear.

In line with its role in cochlear amplification, activation of the efferent system induces a protective effect against noise overstimulation (Handrock and Zeisberg, 1982; Patuzzi and Thompson, 1991; Zheng et al., 1997; Tong et al., 2013; Dinh et al., 2015; Boero et al., 2018). However, in this paper, we provide data supporting new strategies based on cortical activation for preventing repetitive TTS and eventually HHL. Our multisession anodal stimulation protocol clearly avoids threshold shifts after TTS. The neurological basis for such a sustained and reversible decrease in hearing sensitivity is most likely related to the activation of long-term potentiation of Hebbian responses of the circuits involved in MOC activation (i.e., cortical, midbrain, or superior olivary circuits or most probably all of them). Exploiting neural effects of AC repetitive stimulation will enable the development of new strategies for treating diseases with altered hearing sensitivity (hyperacusis) or hearing loss by acoustic overstimulation.

Performing repetitive chronic stimulation of the temporal cortex of patients will, nevertheless, require overcoming two obstacles: developing a non-invasive procedure and deeply stimulating the sulcus of cerebral cortex convolutions. Notwithstanding the difficulties, a new electric stimulation approach based on temporal interfering electric fields has been recently reported (Grossman et al., 2017; Sunshine et al., 2020). This procedure induces deep stimulation through surface electrodes and, therefore, is a promising method for chronic repetitive stimulation in patients, especially considering the results from ongoing experiments in our laboratory.

## Data Availability Statement

The original contributions presented in the study are included in the article/supplementary material, further inquiries can be directed to the corresponding author/s.

## Ethics Statement

The animal study was reviewed and approved by the Comite de Bioetica–University of Salamanca Servicio de Ordenación y Estructura Sanitaria Ganadera. Dirección General de Producción Agropecuaria e Infraestructuras Agrarias Consejería de Agricultura y Ganadería de la Junta de Castilla y León.

## Author Contributions

MM designed the experiments and wrote the manuscript. AC-R, ID, IP, VC, DP-G, and MM performed the experiments and analyzed the data. IP contributed by performing histological methods. ID conducted the quantitative immunocytochemical study and performed the statistical analysis. AC-R and DP-G contributed to the analysis of ABRs. All authors participated in the discussion of the experiments.

## Conflict of Interest

The authors declare that the research was conducted in the absence of any commercial or financial relationships that could be construed as a potential conflict of interest.

## Publisher’s Note

All claims expressed in this article are solely those of the authors and do not necessarily represent those of their affiliated organizations, or those of the publisher, the editors and the reviewers. Any product that may be evaluated in this article, or claim that may be made by its manufacturer, is not guaranteed or endorsed by the publisher.
